# Further Evidence for the Immunosuppressive Activity of Transmembrane Envelope Protein p15E of Porcine Endogenous Retrovirus

**DOI:** 10.3390/ijms27021094

**Published:** 2026-01-22

**Authors:** Joachim Denner, Reinhard Schwinzer, Claudia Pokoyski, Benedikt B. Kaufer, Björn Dierkes, Jinzhao Ban, Lovlesh Lovlesh

**Affiliations:** 1 Institute of Virology, Free University Berlin, 14163 Berlin, Germany; benedikt.kaufer@fu-berlin.de (B.B.K.); bjoern.dierkes@gmail.com (B.D.); jinzhao.ban@fu-berlin.de (J.B.);; 2 Department of General, Visceral and Transplant Surgery, Hannover Medical School, 30625 Hannover, Germany; schwinzer.reinhard@mh-hannover.de (R.S.);

**Keywords:** retroviruse, porcine endogenous retroviruse (PERV), immunosuppression, transmembrane envelope protein, immunosuppressive domain, cytokines, xenotransplantation

## Abstract

Retroviruses are immunosuppressive, and there is evidence that a highly conserved immunosuppressive domain (isu domain) in their transmembrane envelope protein contributes to this activity. Studies have shown that inactivated retroviruses, their purified transmembrane envelope proteins, and synthetic peptides corresponding to the isu domain inhibit mitogen-triggered proliferation of peripheral blood mononuclear cells (PBMCs) and modulate their cytokine and gene expression. This has been demonstrated for human immunodeficiency virus type 1 (HIV-1), as well as for beta- and gammaretroviruses and for both exogenous and endogenous retroviruses, including syncytins. In the case of HIV-1, homopolymers of its isu peptide stimulated an increased release of IL-10, IL-6, and other cytokines from human PBMCs. Up-regulated genes included IL-6, IL-8, and IL-10, as well as MMP-1, TREM-1, and IL-1β. In vivo, in a mouse tumor model, tumor cells that were unable to induce tumors in immunocompetent animals gained the ability to do so when expressing the transmembrane envelope protein or the isu domain of various retroviruses on their surface. Here, we demonstrate that the transmembrane envelope protein p15E of PERV can modulate cytokine expression in human PBMCs. Human 293 cells were transfected with four constructs that express a portion of p15E, including the isu domain, and were cultured in the presence of a selection medium containing hygromycin. The p15E-expressing cells were co-cultured with human PBMCs, leading to the release of IL-6 and IL-10 protein and the modulation of multiple cytokines and other markers, including IL-6, IL-10, IFN-α, TNF-α, MMP1, and SEPP1. Similar, but more pronounced, effects were observed when PERV-producing 293 and pig cells were used in parallel; both expressed higher levels of p15E. Additionally, p15E expression reduced MHC class I expression, and preliminary data indicate that p15E expression could have a protective effect against cellular cytotoxicity. This finding underscores the need for further research to elucidate the dynamics of p15E expression and its immunosuppressive activity. It also contributes to the understanding of the immunosuppressive properties of pathogenic retroviruses. Furthermore, expressing the immunosuppressive p15E of PERV on the surface of a pig xenotransplant may reduce the need for pharmaceutical immunosuppressants.

## 1. Introduction

Retroviruses are immunosuppressive. This is not only true for immunodeficiency viruses such as human immunodeficiency virus 1 and 2 (HIV-1 and -2) but also for most other retroviruses, including gammaretroviruses (for a review, see [1,2]). Feline leukemia viruses (FeLVs), murine leukemia viruses (MuLVs) and koala retroviruses (KoRVs)—gammaretroviruses closely related to porcine endogenous retroviruses (PERVs)—induce in the infected host not only leukemia and lymphoma but also a severe immunodeficiency which usually precedes tumor development. Immunosuppression without tumor development has also been observed.

The mechanism of how retroviruses induce immunosuppression is not well studied. The observation that many retroviruses cause immunodeficiency suggests the existence of shared mechanisms [1]. In the case of lentiviruses, however, additional viral factors clearly contribute to immune dysfunction. For example, the HIV-1 surface envelope protein gp120 binds CD4 on lymphocytes and perturbs immune signaling [3], and the viral transactivator Tat has also been implicated in immunomodulation [4]. Moreover, the accessory protein Nef from both HIV-1 and SIV contributes to AIDS pathogenesis by impairing the humoral and cellular arms of innate and adaptive immunity [5]. Nef down-regulates CD4 and MHC-1. However, gp41 and gp120 are the first viral proteins that interfere with the immune system during the initial step of HIV-1 infection. Nef and Tat are produced only after infection and may contribute to immunosuppression later.

It has been shown that inactivated retroviruses, their transmembrane envelope proteins, or synthetic peptides corresponding to a highly conserved domain in their transmembrane envelope protein, called the immunosuppressive (isu) domain, inhibit different in vitro activities of immune cells, including down-regulating the Th1-type cytokines (interferon-gamma) and up-regulating the Th2-type cytokines (interleukin 10) [1,2]. It is important to note that Nef of HIV-1 contains a sequence homology with the immunosuppressive domain of gammaretroviruses [6]. The isu domain of the transmembrane envelope protein is highly conserved among all retroviruses [1]. The isu motif is not only highly conserved among retroviruses; it is also ancient. The sequence is found in human syncytin-1 and syncytin-2, which are >25 million and >40 million years old, respectively; it is also found in HEMO, another Env protein coded by an endogenous retrovirus that is >100 million years old, and in the percomorf locus of ray-finned fish, which is also >100 million years old [7,8,9,10]. To investigate the function of the isu domain, several mutants with single amino acid substitutions at conserved positions in the isu domain of MuLV were produced [11]. The majority of these mutants abolished infectivity, indicating that such mutated viruses cannot be investigated in vivo [11,12].

The assays used to demonstrate that the retroviral transmembrane envelope proteins and the corresponding isu peptides are immunosuppressive include mitogen-triggered proliferation of PBMCs, mixed lymphocyte reaction, IL-2-stimulated proliferation of T cells, mitogen-triggered proliferation of B cells, neutrophilic and erythroid cell function, receptor motility on the cell surface of immune cells, measurement of cytokine release, and measurement of cytokine and general gene expression (for a review, see [1,2]). A synthetic peptide corresponding to the isu domain of p15E of gammaretroviruses has been shown to inhibit cytotoxic T cell activity, natural killer cell activity, and B cell activation. This peptide inhibits human IFN gamma production and TNF alpha expression. Furthermore, it activates mitogen-activated protein kinases, induces intracellular cAMP, phosphorylates protein kinase D, and inactivates protein kinase C (for a review, see [13,14]). A synthetic peptide with sequence identity to the isu domain of the transmembrane protein gp41 of HIV-1 has also been shown to inhibit protein kinase C [15].

In studies using the recombinant transmembrane envelope protein gp41 of HIV-1 [16,17,18,19,20], it may be assumed in retrospect that the retroviral materials were contaminated with traces of endotoxin, which is also able to induce IL-10 and other cytokines [21,22].

Most importantly, the retroviral transmembrane envelope proteins are also immunosuppressive in vivo. The most convincing in vivo results came from a tumor rejection assay: expression of different retroviral transmembrane envelope proteins on mouse tumor cells, which did not grow in immunocompetent mice, allowed them to produce tumors in immunocompetent animals by suppression of their immune system. This was shown for p15E of MoMuLV [23], the transmembrane envelope proteins of Mason-Pfizer monkey virus [24], human endogenous retrovirus-H (HERV-H) [25], FeLV [26], and one of two murine and one of two human syncytins [27]. Syncytins are envelope proteins of endogenous retroviruses expressed in the placenta [27]. Experiments deleting parts of the isu sequence of syncytin 2 showed that this domain is the sequence responsible for immunosuppressive activity [27]. Only one murine and one human syncytin were immunosuppressive: human syncytin-2 (HERV-FRD) and mouse syncytin-B. In contrast, human syncytin 1 (HERV-W) and murine syncytin-A were not immunosuppressive. Mutations of relevant amino acids in the isu domain allowed for switching from an immunosuppressive syncytin into a non-immunosuppressive and vice versa, as measured in the mouse tumor model [27].

Furthermore, immunization with the non-immunosuppressive form (wild-type syncytin-1 and mutated syncytin-2) induced immunoglobulin G titers 10- to 30-fold higher than the corresponding immunosuppressive form (mutant syncytin-1 and wild-type syncytin-2) [27]. This indicates that the immunosuppressive activity acts not only locally, on the surface of the tumor cells, but is generalized, also influencing antibody production.

Retrovirus infections modulate the cytokine release in the infected individuals, for example, in AIDS patients [28]. The transmembrane envelope proteins and synthetic peptides corresponding to the immunosuppressive domain have also been shown to modulate cytokine mRNA expression and release in human PBMCs. Using cytokine arrays, it was shown that the transmembrane envelope proteins of HIV-1, PERV, HERV-K, and the corresponding isu peptides induced the release of the following cytokines: IL1-α, IL-10, IL-6, IL-8, monocyte chemoattractant protein (MCP)-1, MCP-2, tumor necrosis factor (TNF-α), macrophage inflammatory protein (MIP)-1α, and MIP-3 [29,30,31]. In contrast, the expression of IL-2 and the chemokine (C-X-C motif) ligand CXCL-9 was decreased. Microarray analysis of the expression of more than 25,000 genes in human PBMCs treated with the homopolymer of the HIV-1 isu peptide or with the recombinant transmembrane envelope protein of HERV-K confirmed the cytokine data and showed up-regulation and down-regulation of more than 300 genes [30,31]. Among the genes with the highest up-regulation were IL-6, matrix metalloproteinase 1 (MMP-1), and triggering receptor expressed on myeloid cells 1 (TREM-1). Among the down-regulated genes were ficolin-1 (FCN1), selenoprotein P, plasma, 1 (SEPP1), triggering receptor expressed on myeloid cells 2 (TREM-2), and CXCL-10; all of these proteins are involved in innate immunity [30,31].

The knowledge of the mechanisms of the immunosuppressive activity of retroviruses may have importance for vaccine development against retroviruses: the mutation of the isu domain significantly increased the efficacy of a vaccine against FeLV [26] and against simian–human immunodeficiency virus (SHIV) [32]. Cynomolgus macaques were vaccinated with measles virus replicative vectors expressing antigens of SHIV. Antigens were either the wild type or mutated in the isu domain of the envelope protein. The inactivation of the isu domain led to the induction of significantly enhanced cellular immune responses and reduced proviral loads after challenge of the vaccinees [32]. A mutation in the isu domain of gp41 of HIV-1 increased antibody production when rats were immunized with the mutated protein, in contrast to the unmutated protein [29].

Here, we describe a novel and endotoxin-free system for testing the immunosuppressive properties of a retroviral transmembrane envelope protein. For this, four constructs encoding a part of the transmembrane envelope protein p15E of PERV-A, which is a gammaretrovirus closely related to MuLV, FeLV, and KoRV—all three viruses inducing severe immunodeficiencies in their infected hosts—were transfected into human 293 cells. The transfected cells were continuously cultured in the presence of hygromycin-containing selection medium. Their expression was controlled by immunofluorescence and flow cytometry. These cells were incubated with human PBMCs, and the changes in cytokine release and cytokine expression of the PBMCs in this endotoxin-free system were analyzed in comparison to cells not expressing p15E. In addition, human PBMCs were incubated with pig kidney cells, PK15 cells, releasing PERV, and human 293 cells infected with and producing PERV-A/C [33,34]. Furthermore, p15E-expressing cells were used to study the impact of the expression of p15E on human cytotoxic cells and the expression of the major histocompatibility complex (MHC) class 1 molecules.

## 2. Results

### 2.1. Sequence Homology of the Retroviral Isu Domain

**Figure 1:**
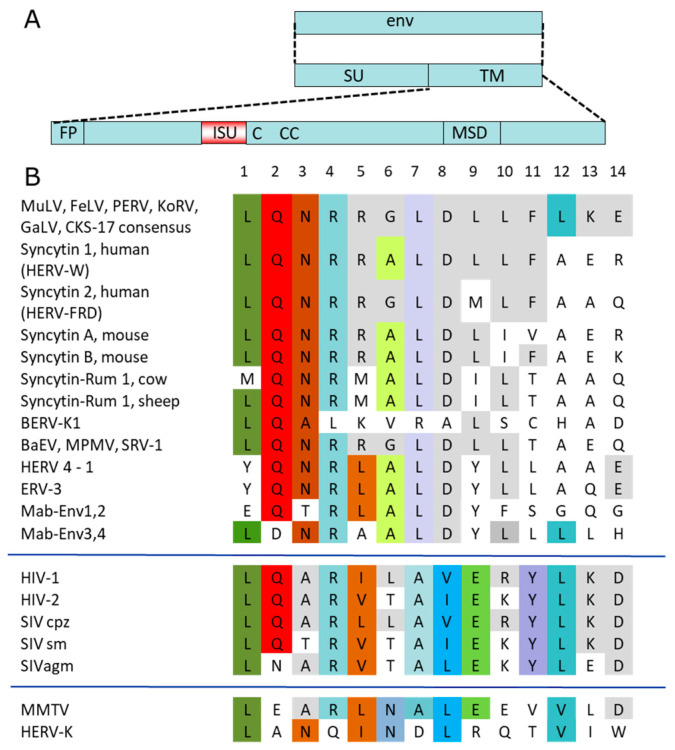
Schematic presentation of retroviral envelope proteins and comparison of the sequences of the immunosuppressive domains of different exogenous and endogenous retroviruses. (**A**) The surface envelope protein (SU) and the transmembrane envelope protein (TM) were transcribed from the envelope gene (*env*). The main domains of the TM protein are the fusion peptide (FP), the immunosuppressive domain (isu), the cysteine loop (C-CC in the case of HIV-1), and the membrane-spanning domain (MSD). (**B**) A sequence alignment of the following retroviral sequences was performed: MuLV, murine leukemia virus; FeLV, feline leukemia virus; PERV, porcine endogenous retrovirus; KoRV, koala retrovirus; GaLV, gibbon ape leukemia virus; CKS-17 consensus sequence of the gammaretroviruses; HERV-W, HERV-FRD, human endogenous retrovirus W, FRD; BERV, bovine endogenous retrovirus; MPMV, Mason Pfizer monkey virus; BERV-P, bovine endogenous retrovirus; BaEV, baboon endogenous retrovirus; HERV4-1, human endogenous retrovirus 4-1; ERV-3, endogenous retrovirus 3; Mab-Env1–4, syncytins of Marubya lizards; HIV-1, -2, human immunodeficiency virus -1, -2; SIV cpz, simian immunodeficiency virus chimpanzee; SIV sm, SIV sooty mangabey; SIVagm, SIV African green monkeys; MMTV, mouse mammary tumor virus; HERV-K, human endogenous retrovirus-K. Identical amino acids and conservative exchanges (L = V = I) in all virus groups or in a single group are stained. The colors highlight identical amino acids at a given position, illustrating the degree of conservation.

A sequence alignment of transmembrane envelope proteins of numerous retroviruses shows that the isu domain is highly conserved among all retroviruses (Figure 1). It is an α-helical structure in immediate neighborhood of a Cys-Cys loop containing three cysteines in the case of gammaretroviruses (CX_6_CC) and only two cysteines in the case of immunodeficiency viruses, including HIV-1 (Figure 1). Within the isu domain, residues L1, Q2, N3, R4, L7, D8, and L10 show the highest conservation.

### 2.2. Cloning and Transfection of p15E of PERV

**Figure 2:**
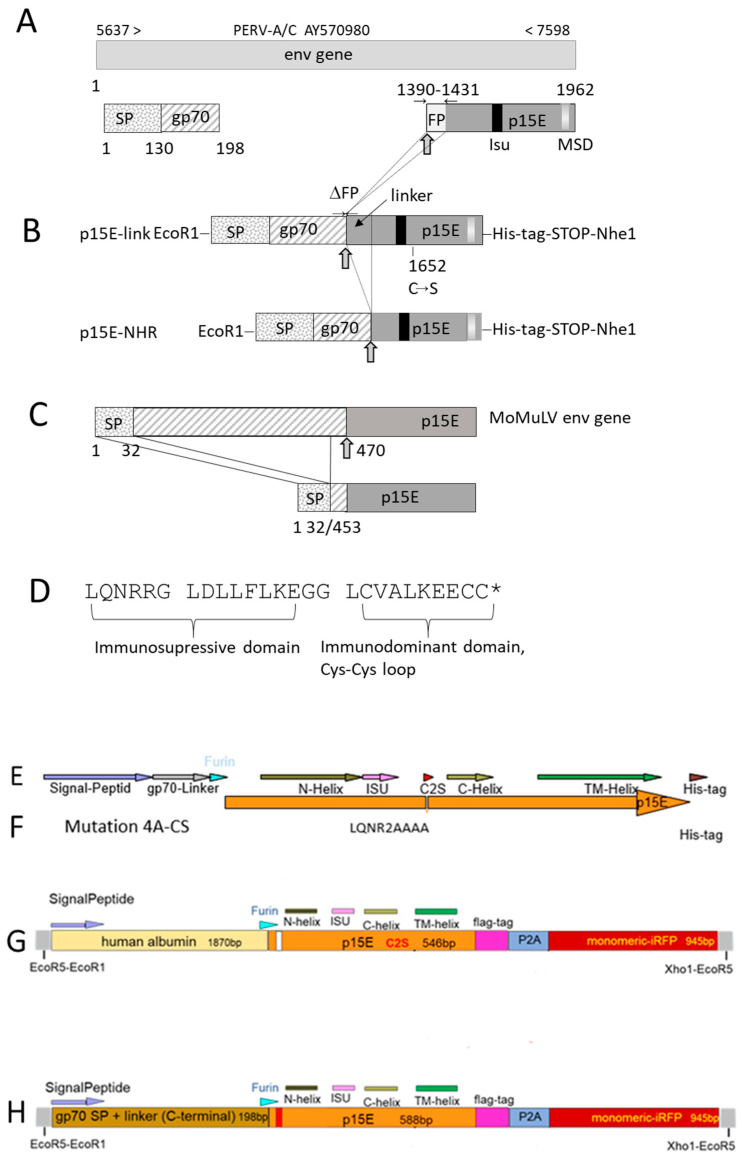
Schematic presentation of the expression constructs of PERV p15E: (**A**) Scheme of the envelope gene (env) used for construction based on the PERV A/C NCBI database entry AY570980. SP, signal peptide; gp70, 23 amino acid residues of the surface envelope protein; FP, fusion peptide of the transmembrane envelope protein p15E sequence; isu, immunosuppressive domain; MSD, membrane-spanning domain. The arrow (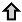) indicates the furin peptidase cleavage site, which is positioned after the carboxy-terminal arginine (Arg) residue in the sequence –Arg–X–Lys/Arg–Arg↓– (where Lys is lysine, X is any amino acid, and ↓ identifies the cleavage site). Numbers above entry indicate the nucleotide according to the PERV sequence; numbers below refer to the nucleotide position according to the env gene. (**B**) Schematic presentation of p15E-link-His and p15E-NHR-His expression constructs. ΔFP, deletion of the fusion peptide coding region; C–S, single-nucleotide exchange at position 1652 that leads to a cysteine-to-serine substitution. (**C**) Sequence of the full-length Moloney murine leukemia virus (MoMuLV) envelope (env) gene, the signal peptide (SP), and the proteolytic cleavage site at position 470 (arrow) are indicated, and the final p15E sequence is expressed in murine tumor cells as described by Mangeney and Heidmann [23]. (**D**) Partial sequence of the transmembrane envelope protein of PERV containing the immunosuppressive and immunodominant domains. * indicates the mutated cysteine in p15E-link. (**E**) This construct corresponds to construct p15E-link-His shown in (**B**). (**F**) The same construct as in (**E**), but with a mutation in the immunosuppressive domain, substituting LQNR into AAAA. (**G**) A new construct, combining human albumin with p15E, flag-tag, and infrared fluorescent protein (iRFP), (**H**) A new construct combining a fragment of the surface envelope protein gp70 of PERV with p15E, flag-tag, and iRFP. iRFP, infrared fluorescent protein; N-helix, N-terminal helix; C-helix, C-terminal helix; TM, transmembrane domain; ISU, immunosuppressive domain; furin, furine peptidase cut; 2AP, 2A peptide.

To establish a cellular and endotoxin-free system for studying the immunosuppressive properties of p15E of PERV, two distinct expression constructs were designed according to the PERV-A/C sequence AY570980 and inserted in a vector. Both constructs contained the ectodomain of p15E, including the immunosuppressive domain, the membrane-spanning domain (MSD), the env signal peptide (SP), the furin peptidase cleavage site, and a short sequence derived from gp70 (Figure 2A,B). Neither construct contained main parts of the fusion peptide (FP) of p15E to avoid unwanted membrane fusions. All constructs were based on the crystal structure of the p15E ectodomain, which forms a homotrimer [35]. One construct, p15E-link, carried a mutation at position 1652, resulting in a cysteine-to-serin substitution, which removed one of the cysteines in the immunodominant region of p15E (Figure 2D) to avoid unwanted intermolecular Cys-Cys interactions. This construct contained a longer portion of the N-terminal part of p15E, referred to as the linker region, to study whether the unstructured region in front of the N-terminal alpha helix of p15E plays a role. Both constructs were similar to the MoMuLV env used to study the immunosuppressive properties of this murine retrovirus in vivo, in a tumor system in mice [23] (Figure 2C). p15E was cloned into pVitro2-EGFP [36]. Following transfection using polyethylenimine (PEI) [37], cells were selected with 500 µg/mL hygromycin and maintained under continuous culture in selection medium.

Since, as shown below, cell surface expression of p15E was very low for these constructs, a second set of constructs was generated. In addition to the p15E-link-His construct, a mutant containing a substitution in the immunosuppressive (isu) domain was produced, in which the sequence LQNR was replaced by AAAA (Figure 2E,F). Previous studies have shown that mutations in this highly conserved region of the isu domain abrogate the ability to induce IL-10 and IL-6 [29].

Furthermore, two additional constructs expressing p15E were generated. One construct expressed human albumin as a protein carrier together with p15E, a FLAG tag, and an infrared fluorescent protein (iRFP) (Figure 2G). The second construct expressed a fragment of the PERV surface envelope protein gp70 together with p15E, a FLAG tag, and iRFP (Figure 2H). The presence of iRFP enabled easy detection of expressing cells and facilitated the selection of positive cells by fluorescence-activated cell sorting (FACS).

### 2.3. Analysis of p15E of PERV Expression in Transfected and Virus-Producing Human Cells

**Figure 3:**
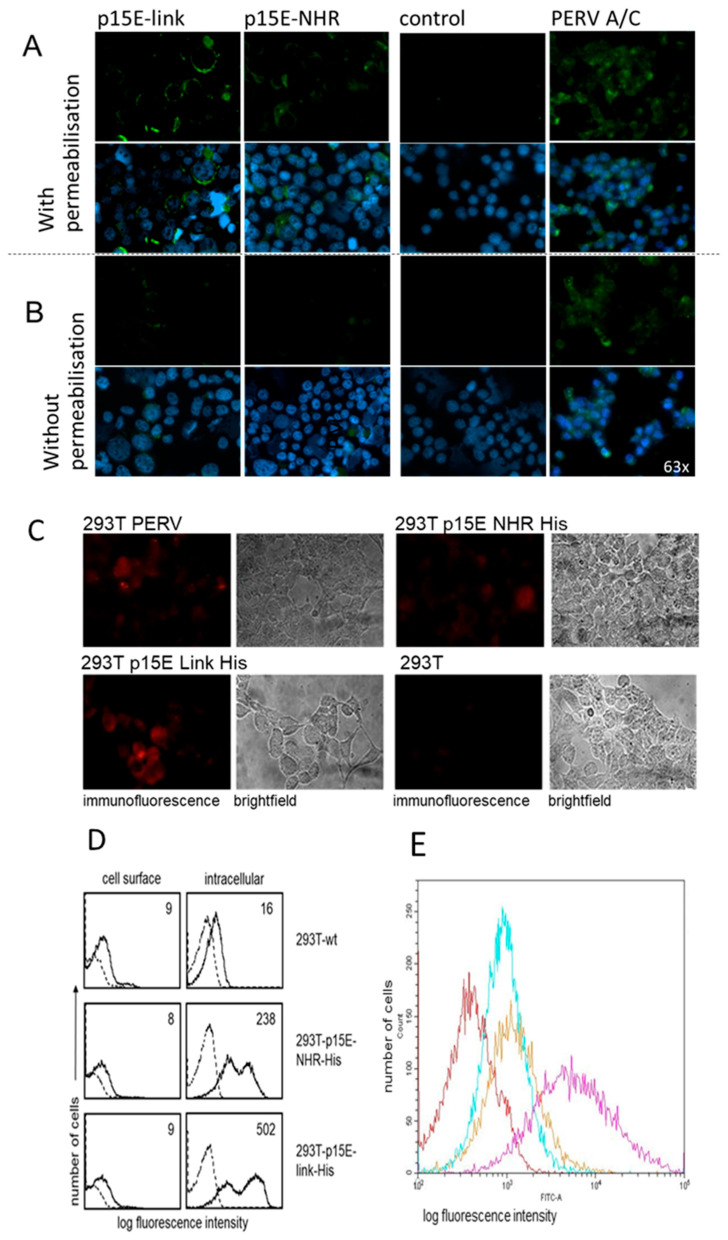
Expression of p15E in transfected and PERV-producing 293T cells. (**A**) Immunofluorescence analysis using a p15E-specific goat antiserum (#355 [38]) and FITC-conjugated donkey anti-goat antibody. The cells were mounted using antifade mounting medium containing 4′,6-diamidino-2-phenylindole (DAPI) of 293 cells transfected with p15E-link and p15E-NHR, of wild-type 293 cells (control) and of 293 cells infected with and producing PERV-A/C. Cell membranes were with permeabilized Triton X-100, showing intracellular expression. Magnification: 63 times. (**B**) Immunofluorescence without permeabilization showing cell surface expression. Magnification: 63 times. (**C**) Immunofluorescence analysis using a p15E-specific goat antiserum (#355) and Alexa 568-conjugated anti-goat IgG of 293 cells transfected with p15E-link and p15E-NHR of wild-type 293 cells (control) and of 293 cells infected with and producing PERV. The cells were not permeabilized to study cell surface expression. Magnification: 63 times; exposure: 3 s; laser: 555 nm. (**D**) Flow cytometry analysis of cell surface and intracellular expression of p15E in wild-type (wt) 293T cells and 293T cells transfected with p15E-NHR-His and 293T-p15E-link-His. Cells were incubated with anti-p15E goat serum #355, followed by incubations with biotinylated bovine anti-goat Ig and APC-conjugated streptavidin. Histograms show anti-p15E binding (solid lines); the numbers represent mean fluorescence intensity and the reactivity of secondary reagents alone (dotted lines). (**E**) Cell surface expression of p15E on 293T-p15E-NHR-His cells (cyan), on 293T-p15E-link-His cells (orange), and PERV-A/C-producing 293 cells (magenta) in comparison with wt 293 cells (red). Flow cytometry analysis was performed using the same antiserum #355 against p15E of PERV and a donkey anti-goat FITC-labeled secondary antibody.

The expression of p15E on the surface of transfected and virus-producing cells was analyzed using two methods: immunofluorescence and flow cytometry analysis. In both cases, a specific antiserum against p15E of PERV was employed. This antiserum (#355) had previously been shown to react with recombinant and viral p15E in Western blot assays, and the epitopes of the antibody binding had been defined using overlapping peptides. GPQQLEK/T is the minor epitope in the fusion peptide proximal region (FPPR) of the N-terminal helix and FEGWFN the major epitope in the membrane proximal external region (MPER) of p15E [38,39]. Low intracellular expression of p15E was observed in the transfected cells when the immunofluorescence was performed with cell membrane permeabilization (Figure 3A). The expression of p15E on the cell surface was even lower. The intracellular expression of p15E was much stronger in virus-producing 293T cells and much stronger on the cell surface compared with the transfected cells (Figure 3). In another experiment assessing p15E expression using the same goat anti-p15E serum (#355) but an Alexa 568-conjugated anti-goat IgG, again, only low levels of p15E were detected, not only in the transfected 293 cells but also in the PERV-producing 293 cells (Figure 3C), indicating that not only does the expression of p15E fluctuate in 293T cells but so does the expression of PERV in PERV-producing 193T cells. It is important to note that the conformation of the protein in both the cytoplasm and on the cell surface remains unknown. p15E on the cell surface might trimerize, and the epitopes recognized by the goat serum may be partially masked.

Flow cytometry studies confirmed significant differences in p15E expression between the cell surface and the intracellular compartment (Figure 3D). Cell surface staining of 293T wt cells with the anti-p15E antiserum revealed a small shift in fluorescence intensity (nine arbitrary units, solid histograms) as compared to incubation of cells with the secondary reagents alone (broken histograms). This shift could be due to some unspecific binding of the antiserum to 293T cells. Fluorescence intensity was not enhanced after staining of 293T-p15E-NHR-His (eight units) or 293T-p15E-link-His cells (nine units), suggesting that p15E was not expressed on the cell surface or with very low density which was below detection level. A similar issue as described above arises here: the conformation of the protein on the cell surface remains unknown. However, clear-cut binding of the anti-p15E antiserum was demonstrated in permeabilized transfectants. Thus, mean fluorescence intensity of 16 units in 293T wt cells significantly increased to 283 and 502 units after staining of 293T-p15E-NHR-His and 293T-p15E-link-His cells, respectively. Thus, the p15E transgene was expressed in this cell model, and the protein was detected intracellularly but was barely detected on the cell surface.

In a second experiment, both 293T-p15E-NHR-His and 293T-p15E-link-His cells, as well as PERV-A/C-producing 293 cells, were stained with the same antiserum against p15E. A donkey anti-goat FITC-labeled secondary antibody was used, along with FACS buffer containing 1% horse serum. In this experiment, clear surface expression of p15E was detected on the transfected 293T cells, with very strong expression observed on the surface of PERV-A/C-producing cells (Figure 3E).

These results indicate that p15E expression can vary, but in some instances, a distinct and detectable expression is evident.

### 2.4. Effect of p15E of PERV on Cytokine Expression in Human PBMCs

**Figure 4:**
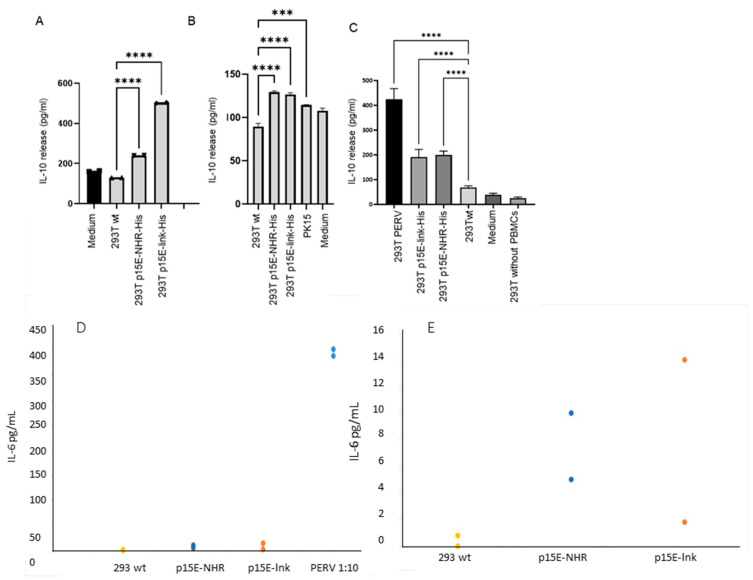
Influence of p15E on IL-10 and IL-6 release by human PBMCs. (**A**) Human 293T cells expressing p15E-NHR-His or p15E-NHR-His, wild-type 293T cells, and medium were incubated with human PBMCs and IL-10 release was measured by ELISA. The marks at the top of the columns indicate the standard deviation. *** means the result is significant at *p* < 0.001, **** means the result is significant *p* < 0.0001. (**B**) In a second experiment, the same cells and, in addition, pig PK15 cells producing PERV were co-incubated with human PBMCs, and IL-10 release was measured. (**C**) In a third experiment, 293T cells producing PERV, 293T cells transfected with p15E-link-His and p15E-NHR-His, 293T wild-type cells, and medium were co-incubated with human PBMCs, and the IL-10 release was measured by ELISA. In addition, 293T cells without PBMCs were tested for IL-10 release, showing that wt 293 cells did not release IL-10. In all experiments, the culture medium containing FCS (designated medium) was tested to exclude FCS batches inducing IL-10 in human PBMCs. Stars indicate statistical significance; statistical analysis was performed using one-way ANOVA. (**D**) Influence of p15E on the IL-6 release by human PBMCs. 293T wild-type cells (293T wt, yellow), 293T cells transfected with p15E-NHR-His (p15E-NHR, blue), 293T cells transfected with p15E-link-His (p15E-lnk, orange), and 293 cells producing PERV-A/C (PERV, blue) were co-incubated with human PBMCs and the IL-10 release was measured by ELISA; results are shown including PERV at a dilution of 1:10. (**E**) The same results, but not including PERV and using a different scale. The two dots each mean a double determination.

To investigate whether 293T cells expressing p15E can induce IL-10 secretion in human PBMCs, similar to the synthetic isu peptides, recombinant transmembrane envelope proteins, and virus preparations of HIV-1 and HERV-K [30,31], p15E-expressing cells were co-incubated with purified human PBMCs. Six experiments were performed. After 24 h, IL-10 levels in the supernatant were quantified using an ELISA. An increase in IL-10 release was observed when PBMCs were incubated with 293T cells expressing both p15E constructs compared to wild-type 293T cells (Figure 4A–C). However, in two of six experiments, no increased expression was observed. Since the expression of p15E on the cell surface was shown to be variable (Figure 3D,E), a correlation with IL-10 release may be anticipated. IL-10 release from wild-type 293T was also tested and, as expected, was undetectable (Figure 4). Human PBMCs incubated with porcine embryonic kidney PK15 cells producing PERV showed a significantly increased IL-10 release (Figure 4B). Notably, a much higher release of IL-10 was observed when PBMCs were incubated with 293T cells producing PERV-A/C (Figure 4C). These cells produced the virus, as demonstrated by measuring viral RNA using a real-time PCR in the supernatant. RNA was isolated from the cell supernatant, and a real-time RT-PCR was performed using the pol primers (Table 1); ct values between 17 and 24 were measured, corresponding to 10^4.7^ to 10^6.4^ copies per 20 µL supernatant. The presence of cellular DNA was excluded performing the assays without RT. Immunofluorescence analysis revealed higher surface expression of p15E on PERV-producing cells compared to that observed in the transfected p15E-expressing cells (Figure 4B). Differences in induced IL-10 levels between the p15E-link-His and p15E-NHR-His constructs observed in some experiments (e.g., Figure 4A) but not in others (e.g., Figure 4B,C), along with the absence of IL-10 induction in two other cases, suggest variability in the expression of active p15E on the surface of transfected cells.

In an additional experiment, the cells analyzed in Figure 3E were co-cultured with human PBMCs, and IL-6 release by the PBMCs was assessed. In contrast to the cells shown in Figure 3D, which exhibited only minimal p15E expression on the cell surface, the p15E-link-His- and p15E-NHR-His-expressing cells displayed low but detectable levels of p15E (Figure 3E). Strong p15E expression was observed on the surface of PERV-A/C-producing cells (Figure 3E). These PERV-producing cells induced robust IL-6 release (Figure 4D), whereas the p15E-link-His-expressing cells and the p15E-NHR-His-expressing cells induced a medium amount of IL-6 (Figure 4E). These findings correlate with the expression levels determined by FACS analysis (Figure 3E) and confirm that the extent of IL-10 release correlated with the extent of p15E on the cell surface.

**Figure 5:**
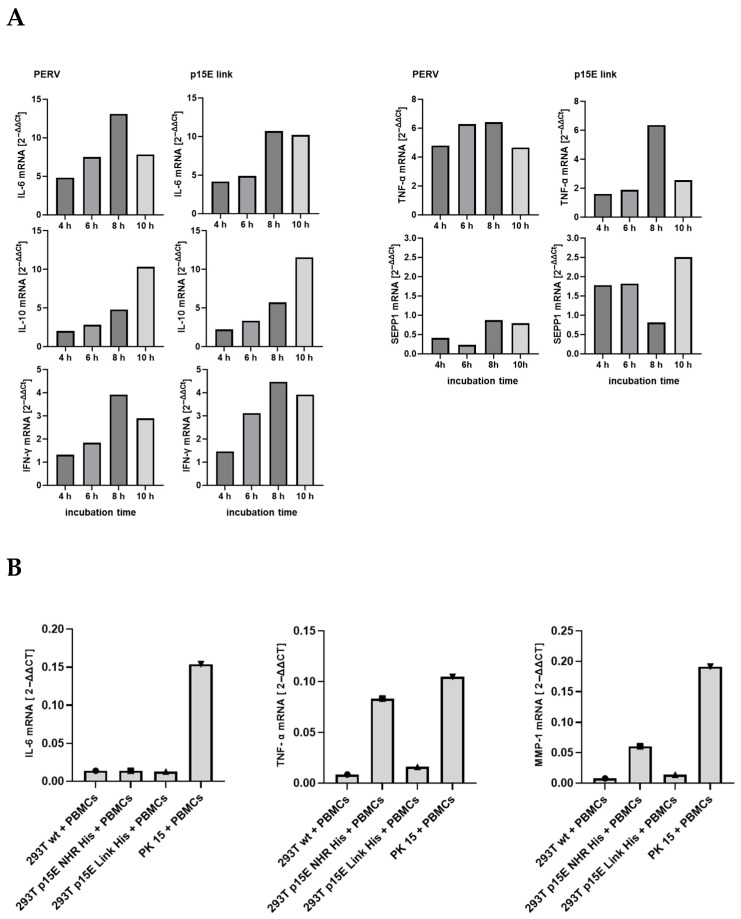
Analysis of cytokine expression at the mRNA level. (**A**) Time course of the influence of PERV-producing 293T cells and 293T cells expressing p15E-link on the expression of different cytokines as well as SEPP1–10 in human PBMCs. PERV-A/C-producing cells (PERV) and 293 cells expressing p15E-link were co-incubated at time point zero with human PBMCs; the RNA was isolated and a real-time PCR was performed to measure the expression of IL-6, IL-10, IFNγ, TNFα, and SEPP1. Human GAPDH was used for normalization. Please note the scale differences, which clearly illustrate the significant variation in expression levels. (**B**) Influence of p15E on cytokine and MMP1 expression. 293T wild-type cells, 293T cells transfected with p15E-link-His and p15E-NHR-His, and PK15 cells were co-incubated with human PBMCs and the expression of IL-6, TNF-α, and MMP1 at the RNA level was analyzed by real-time PCR. Gene expression was calculated according the 2^−ΔΔCt^ method.

After demonstrating that incubation with p15E-expressing cells increased IL-10 and IL-6 protein release by human PBMCs, the effect of p15E on the expression of additional cytokines and markers at the mRNA level was subsequently evaluated. Real-time RT-PCRs specific for the mRNA of IL-6, IL-10, INF-γ, TNF-α, and SEPP1 were established, and the expression was measured after 4, 6, 8, and 10 h of incubation with 293T cells expressing p15E-link-His and 293 cells producing PERV-A/C (Figure 5A). Screening of these cytokines and SEPP1 was performed based on our previous cytokine array and microarray analyses, which showed that these markers were up-regulated in human PBMCs from healthy donors following exposure to the isu peptide homopolymer of HIV-1, as well as the recombinant transmembrane envelope proteins of HERV-K [30,31].

In this experiment, expression of IL-6, IL-10, INF-γ, TNF-α, and SEPP1 mRNA increased, either steadily increasing, as in the case of IL-10, or peaking at 8 h, as in the case of IL-6, TNF-α, and INF-γ. Please note the strong expression of all mRNA in the co-incubation experiment with PERV-infected 293 cells, which showed a higher expression of p15E compared with the transfected p15E-expressing cells. However, when MMP-1, TNF-α, IL-8, and IL-6 were analyzed in a second experiment, an increase in expression of the mRNA of these molecules was only observed for PK15 cells, but not for the transfected cells, with the exception of a slight increase in MMP-1 and IL-6 in cells expressing p15E-link (Figure 5B). Similarly, in this context, the expression of p15E on the cell surface also varies, and a correlation between p15E expression and cytokine expression can be anticipated. Please note again the higher expression of the mRNA in the co-incubation experiment with PERV-producing pig PK15 cells, which showed a higher expression of p15E compared with the transfected and selected p15E-expressing cells. MMP-1 was included because, in microarray experiments with the isu peptide homopolymer of HIV-1 and the recombinant transmembrane envelope proteins of HERV-K, it was identified as the most highly up-regulated gene in human PBMCs [30].

### 2.5. Effect of p15E of PERV on Human Cytotoxic Effector Cells

**Figure 6:**
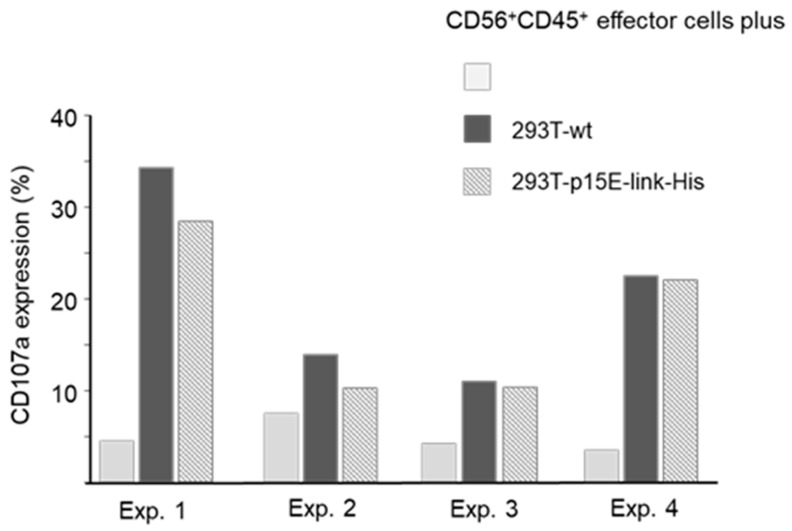
Degranulation of effector cells by co-culturing with 293T cells in four independent experiments (Exp.). IL-2-activated PBMCs were cultured alone (gray columns) or with 293T wt cells (black columns) or 293T cells expressing p15E-link-His (dashed columns). Expression of CD107a was monitored after 2 h on gated CD56^+^CD45^+^ cells.

A specific in vitro assay was applied to evaluate the immunosuppressive effect of p15E of PERV on cytotoxicity of effector cells. Thus, human PBMCs were cultivated for 5 days with IL-2 to induce cytotoxic activity and then co-cultivated for two hours with wild-type 293 cells and 293 cells expressing p15E as p15E-link-His (Figure 6). In a series of experiments using effector populations from different blood donors, 3 to 7% of gated CD56^+^CD45^+^ cells (effector population) expressed CD107a. An increased proportion of CD107a^+^ cells (11 to 34%) was observed in co-cultures with wild-type 293T cells, indicating degranulation of the effector cells by contact with 293T cells. In two experiments (Exp. 1 and 2), we observed a slight reduction in CD107a expression when p15E-expressing transfectants were used as targets. However, no reduction was seen in the other two experiments. This data indicates that under certain conditions expression of p15E on target cells triggers reduced levels of CD107a, pointing to diminished cytotoxic activity of effector cells. This obviously correlates with the level of expression of p15E.

### 2.6. Effect of p15E of PERV on MHC Class I Expression

**Figure 7:**
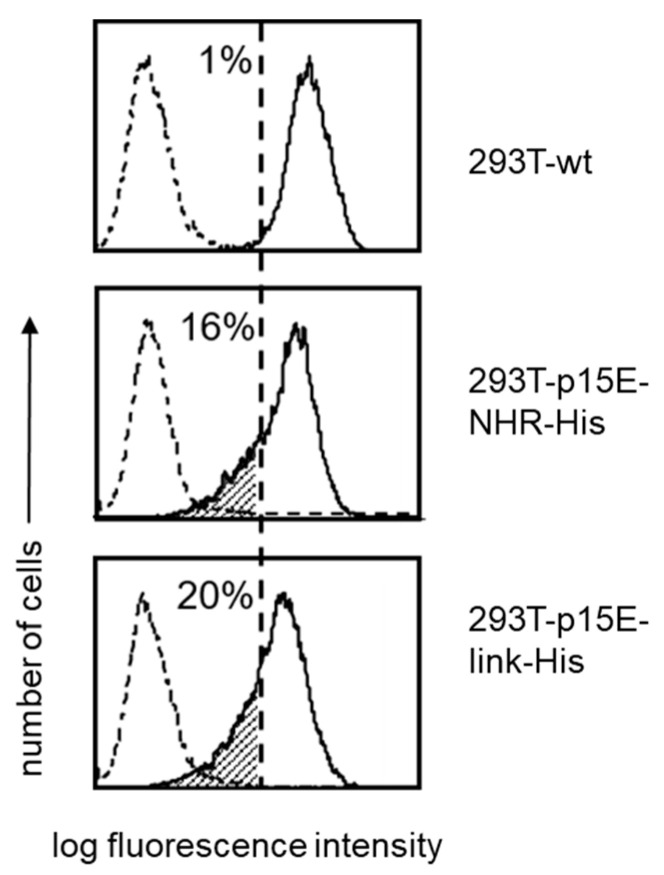
Reduced expression of MHC class-I (HLA-ABC) on p15E-transfected 293T cells. 293T wt, 293T-p15E-NHR-His, and 293T-p15E-link-His cells were stained with a monoclonal antibody to human MHC class-I. The numbers represent percentage of cells carrying reduced levels of MHC class-I molecules (dashed area). Broken-line histograms were obtained after incubation of cells with secondary reagents alone. Shown is one representative experiment out of a series of three.

Retroviruses are known to down-regulate MHC molecules at the cell surface. For example, HIV-1 reduces the expression of MHC class I A and B molecules, thereby shielding infected cells from cytotoxic T lymphocyte (CTL)-mediated killing [42]. A similar effect has been reported for a murine gammaretrovirus closely related to PERV [43]. To examine whether p15E expression affects MHC class I (HLA-ABC) levels, 293T wild-type cells and cells expressing p15E either as p15E-NHR-His or p15E-link-His were stained with a monoclonal antibody against human MHC class I molecules. A significant reduction of 16–20% in MHC class I expression was observed (Figure 7).

### 2.7. New Set of Expression Constructs, Transfection, and Co-Cultivation with Human PBMCs

**Figure 8:**
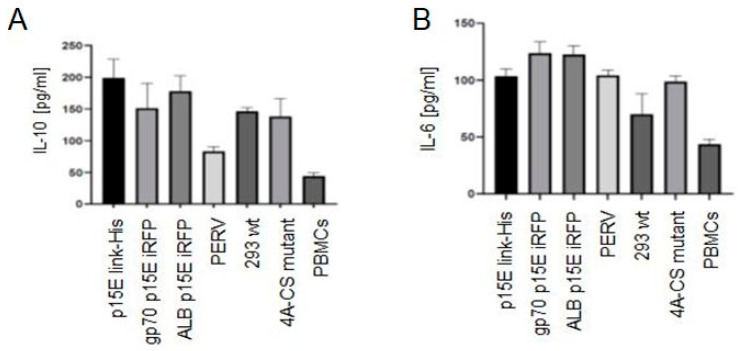
ELISA analysis of IL-10 and IL-6 release by normal human PBMCs after co-incubation. (**A**) IL-10 release after incubation of human PBMCs with 293T cells expressing p15E-link-His (construct Figure 2E); expressing ALB p15E iRFP (construct Figure 2G); expressing gp70 p15E iRFP (construct Figure 2H); and 293 cells producing PERV. Wild-type 293 cells (293 wt) served as control. In addition, 293 cells expressing the 4A-CS mutant with a mutation in the isu domain in the construct p15E link-His (construct Figure 2F) and human PBMCs alone were used. One of three experiments is presented. (**B**) IL-6 release after incubation of human PBMCs with the same cells as in (**A**).

Because p15E expression was very low when the constructs p15E NHR His and p15E Link were used (Figure 4), new expression constructs were generated. First, a mutation was introduced into the isu domain (LQNR → AAAA), which in HIV-1 is known to abolish immunosuppressive activity [29] (Figure 2F). Second, p15E was co-expressed with an infrared fluorescent protein (iRFP): in one construct together with human serum albumin and in another with a larger fragment of the PERV surface envelope protein gp70 (Figure 2G,H). These constructs were transfected into 293T cells using polyethylenimine (PEI), and cells were selected by FACS. However, the p15E expression in this set of constructs also remained very low. Nonetheless, incubation of these p15E-expressing 293T cells with normal human PBMCs resulted in an increased IL-6 and IL-10 release compared with untransfected 293T cells (Figure 8). Importantly, the isu domain mutation reduced the amount of IL-10 released (Figure 8). Interestingly, IL-10 induction by cells producing PERV-A/C was very low in this experiment, consistent with the low level of PERV expression observed in some assays (Figure 3C). This indicates that the expression of p15E and PERV fluctuated in human 293T cells for reasons that remain unknown. The extent of cytokine induction depended on the level of p15E expression in both p15E-expressing and PERV-producing cells.

## 3. Discussion

To gain further evidence for the immunosuppressive properties of the transmembrane envelope protein p15E of PERV, the main part of this molecule, including the isu domain, was expressed in human 293T cells, and its effect on human PBMCs was investigated. Unfortunately, the protein expression on the cell surface was very low and fluctuating, leading to variability in its effects across experiments. Since the expression of p15E was the only parameter fluctuating in the experiments, the modulation of the IL-10 and IL-6 release and cytokine expression found in some experiments must be associated with this molecule. It is important to note that the release of IL-10 and IL-6 (Figure 4) and the expression of cytokine mRNA (Figure 5) were high in the co-incubation experiments with PERV-infected 293 cells and PERV-producing pig PK15 cells, which showed a higher expression of p15E compared with the transfected p15E-expressing cells (Figure 3).

It remains unclear why the expression of p15E, especially on the cell surface of the transfected and selected p15E-expressing 293 cells, is so low. Surprisingly, an arginine repeat was found in the protein sequence of p15E of PERV, while it was absent in the sequence of p15E of MuLV [44]. This short arginine repeat suggests that the PERV protein could be retained in the cell, in contrast to the MuLV protein p15E [44]. Arginine/serine-rich proteins are mainly localized in the cytoplasm and are targeted to the nucleus [45,46]. Future investigations will focus on optimizing the expression system to achieve higher and more stable levels of p15E expression. This will include introducing mutations in the arginine repeats, testing alternative vectors, promoters, and tagged constructs, as well as evaluating trimeric p15E and different cell lines to improve protein expression, stability, and conformation. Nevertheless, few p15E molecules can be found at the surface of the transfected cells by immunofluorescence (Figure 3). Expression of p15E was also found by flow cytometry (Figure 3E). Therefore, two methods, immunofluorescence and flow cytometry, independently showed low expression in the cytoplasm of human 293 cells and a lower but detectable expression at the cell surface. Unfortunately, the expression of p15E on the cell surface fluctuates (see Figure 3D,E); the reasons for this are unclear.

Despite the low expression of p15E, cytokine expression and cytokine release from PBMCs of healthy humans were modulated in a manner consistent with previous observations for synthetic peptides corresponding to the isu domain of PERV p15E and purified PERV particles. Furthermore, previous findings showed that peptides corresponding to the isu domain of PERV inhibited mitogen-triggered proliferation [47,48]. The sequence of the isu domain of PERV is identical to the isu domains of related gammaretroviruses, such as MuLV, FeLV, and KoRV (Figure 1). Therefore, theoretically, evidence of immunosuppressive properties in synthetic peptides, viral or recombinant p15E, or virus particles from MuLV, FeLV, and KoRV inherently extends to the isu domain of PERV, and vice versa. The immunosuppressive properties of MuLV, FeLV, and KoRV, as well as of human endogenous retroviruses such as HERV-K, are well studied in vitro and in vivo (for a review, see [1,2,49]). The envelope proteins of endogenous retroviruses, called syncytins, not only play a role in the placentogenesis but may also immunoprotect the embryo (for a review, see [50]). However, an involvement of PERV in pig placentogenesis has not yet been demonstrated.

Two main reasons may account for the limited effect of the p15E constructs used in this work. The first is its low level of expression, and the second is the possibility that it does not adopt the correct conformation required to bind to the putative receptor for the immunosuppressive domain. Our studies on peptides suggested that a certain degree of multimerization is necessary, as monomeric peptides were inactive, while only polymeric forms exhibited activity [51]. When we displayed the isu domains of HIV, PERV, and MuLV on the surface of human cells using a tetraspanin-anchored construct, no modulation of cytokine release was observed in co-cultured human PBMCs [44], likely due to an unfavorable conformation caused by proximity to the cell membrane. Similarly, when the same retroviral proteins containing the isu domains were expressed in an alternative system and released into the culture medium, no cytokine response was detected, possibly due to insufficient protein levels or lack of multimerization.

Immunosuppression is a general property of all retroviruses, and immunodeficiency viruses such as human immunodeficiency viruses HIV-1 and HIV-2 are well studied examples (for a review, see [1]). The changes in cytokine expression observed here are in agreement with the changes in cytokine expression observed when human PBMCs were incubated with polymers of synthetic peptides corresponding to the isu domain of HIV [31] or with HERV-K particles released from a human teratocarcinoma cell line, with a recombinant transmembrane envelope protein of HERV-K or with peptides corresponding to the isu domain of HERV-K [30]. Modulation of cytokine expression was also observed when FeLV was analyzed (for a review, see [2]).

Analysis of gene expression in human PBMCs treated with the HIV isu peptide or the recombinant transmembrane envelope protein of HERV-K revealed significant changes, with over 300 genes either up- or down-regulated [30,31]. Notably, IL-6, IL-10, MMP-1, and SEPP1 were among the most highly up-regulated genes—a finding that is consistent with the elevated expression levels observed in this study (Figure 4). MMP-1 is a zinc-dependent protease essential for the breakdown of extracellular matrix expressed on monocytes and macrophages [52], and SEPP1 plays an important role in innate immune responses [53,54]. In hepatitis C virus infection, SEPP1 mRNA inhibits type I interferon responses by limiting the function of retinoic-acid-inducible gene I (RIG-I), a sensor of viral RNA [54].

One advantage of the established system is the absence of endotoxin. Endotoxin can induce cytokine modulation [55], and in experiments with synthetic peptides or recombinant proteins produced in bacteria, endotoxin contamination below the detection limit of the assay used (EndoLISA System, Hyglos, Bernried, Germany) could not be ruled out. Endotoxin is a lipopolysaccharide (LPS) of the outer membrane of most Gram-negative bacteria; it binds first to the LPS-binding protein (LBP) and is transferred to cluster of differentiation 14 (CD14), where myeloid differentiation-2 protein (MD-2) and the Toll-like receptor 4 (TLR4) re-associate. Receptor binding leads to a signal transduction involving activation of the nuclear factor-kappa B (NF-κB) transcription factor, resulting in the release of cytokines [55]. To avoid endotoxin contamination, in our later experiments, gp41 produced in human 293 cells was used [56]. The secreted and purified to homogeneity recombinant gp41 produced in 293 cells was soluble, glycosylated, and assembled into trimers. The protein bound to monocytes and to a lesser extent to lymphocytes and triggered the production of specific cytokines when added to normal PBMCs [56], confirming that endotoxin was not involved in the effects of gp41 of HIV-1.

In addition, the immunosuppressive properties of the transmembrane envelope protein gp41 of HIV-1 was studied in a cellular system which was endotoxin-free. For this, murine cTRAMP prostate cancer cells were transfected with a gp41-expressing vector, and gp41 expression on the cell surface was demonstrated by FACS analysis, and the cells released gp41 into the cell supernatant [56]. These cells were pulsed with the ovalbumin-derived MHC-I peptide SIINFEKL and co-cultured with naïve CD8+ T cells from OT-1 mice, which carry the corresponding SIINFEKL T cell receptor. The gp41-expressing cells, but not the vector control cells, strongly inhibited IFNγ production and reduced CD25 (IL-2 receptor) expression. These findings indicated that gp41 impairs the antigen-specific response of murine CD8+ T cells by drastically suppressing IFNγ production. Furthermore, this result corroborates previous findings that retroviral transmembrane proteins or peptides corresponding to their isu domain exhibit interspecies reactivity by modulating immune cells across species (for a review, see [1]).

Since the modulation of cytokine expression by p15E of PERV observed in our experiments is identical to that reported for FeLV, which contains the same isu domain [26], and comparable to that induced by the transmembrane envelope protein gp41 of HIV-1 [16,17,18,19], which contains a related isu domain, this can be considered a common property of retroviral transmembrane envelope proteins. This conclusion is further supported by findings that synthetic peptides corresponding to the isu domains of PERV, MuLV, FeLV, and KoRV, as well as peptides derived from HIV-1, inhibit mitogen-induced proliferation of human PBMCs and modulate cytokine and gene expression in a similar manner [1,2].

The results presented here, together with previous publications, not only contribute to the understanding of the mechanisms by which pathogenic exogenous retroviruses such as MuLV, KoRV, FeLV, and HIV and endogenous retroviruses such as HERV-K and syncytins exert their immunosuppressive effects but may also have important implications for xenotransplantation. Xenotransplantation using pig cells or organs has achieved remarkable progress in recent years. Notably, the first human patients have received encapsulated pig islet cells for the treatment of diabetes [57], as well as pig hearts [58,59] and kidneys [60] for the treatment of organ failure. Although the pig organs were genetically modified to prevent hyperacute rejection and to reduce cell and antibody mediated rejection, they still required intensive pharmacological immunosuppression to prevent rejection. The expression of an immunosuppressive protein on the surface of the pig transplant may help prevent rejection, similar to what has been demonstrated in mouse tumor models [23,24,25,26,27,61]. In contrast to the mouse tumor model, where expression of the immunosuppressive retroviral transmembrane protein prevents rejection of tumor cells, in xenotransplantation, this mechanism aims to prevent rejection of a healthy transplanted organ. This approach could significantly reduce the need for pharmacological immunosuppression. The p15E of PERV is the best candidate since it is present in the genome of all pigs, and pigs are tolerant and do not produce antibodies against p15E [62]. The expression of the immunosuppressive molecule p15E on the surface of pig xenotransplants could revolutionize the field of transplantation. Despite the xenogeneic origin of these organs, the need for immunosuppressive drugs may be significantly reduced. Additionally, pig organs offer the advantage of being available in unlimited supply and present a higher level of virological safety compared to allogeneic human organs. Whereas donor pigs for xenotransplantation can be screened carefully before transplantation, historically, several human viruses—including HIV, human cytomegalovirus, and even rabies virus—have been transmitted through human organ transplants [63].

## 4. Materials and Methods

### 4.1. Wild-Type 293 Cells, PERV-Producing Human 293 Cells, and Pig Cells

**Figure 9:**
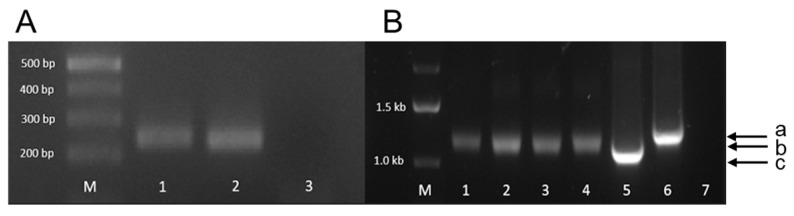
Agarose gel electrophoresis of the PCR amplicons of PERVs integrated in 293 cells. (**A**) Amplification of the *pol* sequence, indicating the presence of the provirus in the cell genome. M, marker. 1, PERV-A/C used in the experiment; 2, positive control; 3, negative control. (**B**) Amplification of the LTR sequence. M, marker. 1–4, amplicons of PERV-A/C used in the experiment (amplicon size b); 5, amplicon of PERV/3° plasmid (amplicon size c); 6, amplicon of PERV/5° plasmid (amplicon size a); 7, negative control.

Human kidney epithelial 293T cells, 293T cells infected with and producing PERV-A/C, and porcine embryonic kidney pig PK15 cells were grown in Dulbecco Eagle Medium (DMEM) with 10% fetal bovine serum (FCS, PAN Biotech, Aidenbach, Germany, Lot P160616) and 1% penicillin–streptomycin (DMEM culture medium). Cells were maintained at 37 °C in a humidified chamber with 5% CO_2_. PK15 cells harbor PERV-A and PERV-B but not PERV-C proviruses in their genome; they release infectious virus particles. These cells were obtained from Leibniz-Institute DSMZ (Deutsche Sammlung von Mikroorganismen und Zellkulturen, Braunschweig, Germany). The PERV-A/C produced by 293T cells is the result of passaging cell-free virus on human 293T cells and is characterized by multimerized repeats containing transcription factor binding sites in the long-terminal repeat (LTR) [33,34]. Thus, a higher number of repeats results in a longer LTR and, consequently, a higher viral replication rate [34]. Since the number of repeats in the LTR changes during cultivation [33], a PCR was performed using LTR-specific primers in order to characterize the virus used in the present experiments. The length of the amplicon was higher compared with that from PERV-3° and lower compared with PERV-5° [33] (Figure 9), indicating that a highly replicating virus is produced.

The virus load was determined using a real-time PCR (see below). 293T cells were split twice a week in a 1:3 ratio; PK15 cells were split in a 1:2 ratio every 3 days after washing with phosphate-buffered solution (PBS) and trypsinization using 0.25% trypsin/0.02% EDTA (PAN Biotech, Aidenbach, Germany).

### 4.2. Cloning of p15E

Synthetic p15E constructs were produced based on the *env* gene of PERV-A/C (AY570980) as gene blocks (gBlock) (Integrated DNA Technologies IDT, Coralville, IA, USA) (Figure 2). All constructs contained the signal peptide of the *env* gene and a linker part of the gp70 gene coding for its first 23 amino acids, followed by the sequence for the furin cleavage site and a modified sequence of the p15E gene. All constructs did not contain a major part of the fusion peptide (position 1390–1431). The constructs of p15E were based on the crystal structure the p15E ectodomain which form a homotrimer, pdb_00007s94 [35]. The reported trimer formation was achieved on the basis of the overexpressed and purified ectodomain, expressed in a bacterial expression system. In our mammalian expression system, the construct is targeted to the plasma membrane and has to be processed by the plasma membrane-located peptidase furin to obtain a final product which is similar to the p15E protein expressed when PERV is produced. Expecting that the length of the N-terminal part might be important for the correct conformation, two versions of difference length were produced. The fusion peptide was removed in both constructs to avoid unwanted membrane fusion.

The construct designated p15E-link contained the following modifications: a nucleotide exchange at position 1652 from g to c (leading to a cysteine-to-serin conversion to avoid unwanted intermolecular Cys-Cys interactions). The construct designated p15E-NHR did not contain the sequence coding for the unstructured N-terminal part, including the fusion peptide, but started with the N-terminal helix (NHR) (nucleotide position 1456) of p15E; the nucleotide at position 1652 was not changed. For cloning purposes, an Eco-R1 restriction site was added to the 5′end, including a Kozak sequence for optimal translation initiation. The 3′end contained a sequence coding for a 6× histidine tag (p15E-link-His, p15E-NHR-His) and a stop codon followed by a Nhe-1 cutting site. The p15E gBlock with Eco-R1 and Nhe-1 site were cloned into pVitro2-EGFP [36] using the same two enzymes replacing the EGFP gene in the plasmid. All plasmids were sequenced before transfection into 293T cells.

The constructs were similar to the construct of the envelope of the Moloney MuLV (MoMuLV) prepared by Mangeney and Heidman [23] (Figure 2C). The immunosuppressive domain of PERV and MoMuLV are identical (Figure 1). The MoMuLV env construct was expressed in mouse cells that do not produce tumors in immunocompetent mice. However, when Env was expressed on the cell surface, tumors developed, indicating that the retroviral protein induced immunosuppression [23].

In addition, a second set of p15E-expressing constructs was generated. Besides the p15E-link-His construct, a mutant carrying a substitution in the isu domain was produced, in which the sequence LQNR was replaced by AAAA (Figure 2E,F). Previous studies have shown that mutations in this highly conserved region of the isu domain abrogate the ability to induce IL-10 and IL-6 [29].

Furthermore, two additional constructs expressing p15E were generated. One construct expressed human albumin as a protein carrier together with p15E, a FLAG tag, and an infrared fluorescent protein (iRFP) (Figure 2G). The other construct expressed a fragment of the PERV surface envelope protein gp70 together with p15E, a FLAG tag, and iRFP (Figure 2H). The presence of iRFP enabled easy detection of expressing cells and facilitated the selection of positive cells by FACS.

### 4.3. Transfection of p15E and Establishment of Transfected p15E-Expressing Cells

For plasmid transfection, 10^5^ 293T cells were seeded in a 12-well plate the day before transfection. On the next day, the medium was changed to DMEM with 5% FCS. For each transfection, 3 µL of polyethylenimine (PEI) solution (1 mg/mL) was added to 50 µL PBS; in parallel, 1 µg plasmid DNA was added to 50 µL PBS [37]. Both solutions were vortexed at high speed for 1 min. After 10 min rest at room temperature, the PEI and DNA solution were gently mixed and incubated for 3 min at room temperature. The transfection solution was added dropwise to the cells. After 3 h, the medium was changed to DMEM culture medium. Selection was started 2 days after transfection with 500 µg/mL hygromycin, and the cells were maintained under continuous culture in the selection medium containing hygromycin.

The iRFP-expressing cells were selected by FACS.

### 4.4. Peripheral Blood Mononuclear Cells (PBMCs)

At the Institute of Virology in Berlin, PBMCs were isolated from buffy coats from human blood from an anonymous donor using Ficoll-Hypaque density centrifugation with the use of 50 mL Leucosep Tubes (Greiner Bio-One, Kremsmünster, Austria) according to the instructions of the manufacturer (Greiner Bio-One). The buffy coat was diluted in a 1:2 ratio with PBS beforehand. Leucosep tubes were filled with 15 mL of Ficoll-Hypaque and centrifuged for 30 s at 1000× *g* at room temperature to move the Ficoll-Hypaque below the porous barrier. A volume of 30 mL of the diluted buffy coat was layered on top of the porous barrier and centrifuged at 1000× *g* for 10 min at room temperature without brakes. After centrifugation, the following layers were observed: plasma, enriched cell fraction PBMCs, granulocytes, and erythrocytes. The fraction containing PBMCs was harvested using a Pasteur pipette. The porous barrier effectively avoids recontamination with pelleted erythrocytes and granulocytes. Harvested PBMCs were washed twice with 10 mL of PBS and subsequently centrifuged for 10 min at 250× *g*. The PBMC pellet was resuspended in cell culture medium. Resuspended PBMCs were counted using a Neubauer Chamber, and 1 × 10^8^ PBMCs were frozen in cryopreserved tubes and stored in liquid nitrogen in a freezing medium containing 70% DMEM, 20% FCS, and 10% dimethyl sulfoxide (DMSO). Freshly isolated PBMCs were used for co-culture experiments. The use of human blood has been approved by the ethical commission at the Medical Faculty of Humboldt University Berlin. At the Transplant Laboratory in Hannover, PBMCs were isolated from discarded material of normal routine apheresis samples from anonymized donors.

### 4.5. Ethics Declarations

At the Institute of Virology in Berlin, buffy coats from human blood from an anonymous donor were provided by Deutsches Rotes Kreuz, Blutspendedienst Nord-Ost, Berlin. The use of human blood has been approved by the ethical commission at the Medical Faculty of the Humboldt University Berlin. At the Transplant Laboratory in Hannover, PBMCs were isolated from discarded material of normal routine apheresis samples obtained from the Department of Transfusion Medicine of the Hannover Medical School. Samples were anonymized and could not be assigned to an individual donor. The local ethics committee of Hanover Medical School approved this procedure. All methods were carried out in accordance with DFG guidelines of Good Scientific Practice.

### 4.6. Co-Cultivation

Human 293T cells and porcine PK-15 cells are adherent cells and were used in the co-cultivation assay at 80 to 90% confluence. Cells were washed with PBS and incubated with 0.25% trypsin/0.02% EDTA at 37 °C for 30 s. A volume of 10 mL of culture medium containing FCS was added; cells were collected in a 15 mL falcon tube and centrifuged at 300× *g* for 5 min. Pellets were resuspended in 10 mL culture medium. Cells were counted twice using a Neubauer Chamber and centrifuged at 300× *g* for 5 min, and culture medium was added to the cell pellet to obtain 3 × 10^5^ cells/100 μL. A total of 100 μL of cells was added to each well of a 96-well plate, and 100 μL of culture medium without hygromycin was added into the wells. Cells were incubated overnight at 37 °C in a humidified chamber with 5% CO_2_. The next day, 100 μL of media was removed, as 293T and PK-15 adhere to the surface of the plate, and 3 × 10^5^ PBMCs in a volume of 100 μL RPMI with 15% FCS, 2 mM glutamine, and 1 mM sodium pyruvate were added for co-incubation and left overnight at 37 °C in a humidified chamber with 5% CO_2_. For PCR expression studies, pooled batches from wells with 2.5 × 10^4^/100 µL 293T cells and 7.5 × 10^4^/100 µL PBMCs were used.

### 4.7. Fluorescence Analysis

293T cells were gently dislodged from cell culture flasks with ice-cold PBS and counted with a Neubauer Chamber after trypan blue staining to detect dead cells. The cell suspension was adjusted to 250 × 10^3^ cells/mL. For each sample, 50 × 10^3^ cells in 200 µL PBS were transferred to a glass slide using a Cellspin 1 device (Tharmac, Limburg/Lahn, Germany) at 8000 rpm for 10 min according to the manufacturer’s instructions. Slides were kept at room temperature until completely dry. Cells were fixed in 4% formaldehyde in PBS for 15 min followed by washing in PBS three times for 10 min each. Perforation of the cell membrane was achieved by 15 min incubation in PBS with 0.5% Triton X-100 (Carl Roth, Karlsruhe, Germany) followed by PBS washes as described above. Slides were incubated with 3% BSA in PBS for 1 h to reduce unspecific binding followed by incubation with a 1:100 dilution of goat anti-p15E (goat #355 [38]) in 3% BSA/PBS for 1 h. After the PBS wash (3 times, 10 min) FITC-conjugated anti-goat antibody from a donkey (Merck/Sigma Aldrich, Darmstadt, Germany) was incubated for 1 h at room temperature. Cover slips were added after all liquid was removed, and a mounting dye containing 4′,6-diamidino-2-phenylindole (DAPI) was added (Carl Roth, Karlsruhe, Germany).

Samples were analyzed with a Zeiss Axio fluorescence microscope equipped with an Axiocom 503 mono camera and a Colibri 7 LED light source using ZEN 2.3 software (all Zeiss, Oberkochen, Germany). The SMART set up provided by the software was used to adjust the fluorescence signals. Exposure time for the FITC channel was set to 5 s (DAPI 50 ms). Non-specific background signals in the FITC channel were subtracted by setting the threshold in the negative control (untransfected 293T cells) to zero/black. These setting were used for all samples. DAPI staining was adjusted to highest contrast.

### 4.8. Antibodies and Flow Cytometry

A goat anti-p15E serum was used to monitor p15E expression after transfection of human 293T. Generation and characterization of the goat anti-p15E serum #355 had been described previously [38]. Cells were incubated with serum (30 min, 1:40 dilution), followed by two additional incubation steps using biotinylated bovine anti-goat Ig (Dianova, Hamburg, Germany) and allophycocyanin (APC)-conjugated streptavidin (BD Biosciences, San Jose, CA, USA). To monitor intracellular levels of p15E, transfected and control cells were fixed with 4% paraformaldehyde for 10 min at room temperature, permeabilized with saponin (0.2%) for 10 min at room temperature, and then incubated with anti-p15E antiserum. Expression of MHC class I molecules (HLA-ABC) on 293T cells was detected by indirect staining using monoclonal antibody (mAb) W6/32 (American Type Culture Collection, ATCC, Manassas, VA, USA) and phycoerythrin (PE)-conjugated rat anti-mouse kappa light chain (BD Biosciences). Directly labeled mAb CD56-APC (B159; BD Biosciences) in combination with CD107a-PE (H4A3; BD Biosciences) were used to study degranulation of human natural killer (NK) cells in response to 293T cells. An antibody against CD45 was used: CD45-FITC (HI30, BD Biosciences). Analyses were performed on a FACSCalibur flow cytometer (Becton Dickinson, San Jose, CA, USA), and data were processed by using FCS Express 7 (De Novo software, Pasadena, CA, USA). These analyses were performed at the Medical School, Hannover.

An additional assay was performed at the Free University Berlin with the same antiserum against p15E and a donkey anti-goat FITC-labeled secondary antibody, using a special buffer composed of phosphate-buffered saline (PBS), 2 mM ethylenediaminetetraacetic acid (EDTA), and 1% horse serum. Analyses were performed on a Cytoflex S Flow Cytometer (Beckman Coulter, Krefeld, Germany) and data were processed by using Software CytExpert 2.1.

### 4.9. CD107a Assay

Cytotoxic effector cells were generated by culturing PBMCs for 5 to 7 days in the presence of 50 ng/mL IL-2. Cells (2 × 10^5^) were co-cultured for two hours with 2 × 10^5^ 293T wt cells or 293T cells transfected to express p15E (293T-p15E-NHR-His or 293T-p15E-link-His). Cytotoxic activity against 293T target cells was monitored by assessing CD107a expression (degranulation) on gated CD56^+^ CD45^+^ NK cells.

### 4.10. Statistical Analysis

Statistical analyses at the Transplant Laboratory in Hannover were performed using Student’s *t*-test, with levels of significance reported as *p*-values. At the Institute of Virology in Berlin, statistical analysis was performed using one-way ANOVA and using GraphPad Prism (Prism 10.2.2).

### 4.11. DNA and RNA Extraction

In order to demonstrate the presence of the sequence encoding p15E with its isu domain, the cell lines wt 293T, 293T-p15E-NHR-His and 293T-p15E-Link-His were tested using conventional PCR. Genomic DNA was isolated from transfected and non-transfected cells using DNA Easy Blood and Tissue Kit (Qiagen, Hilden, Germany). A total of 5 × 10^6^ cultured cells of each cell line were centrifuged for 5 min at 300× *g* and resuspended in 200 μL PBS. Further steps of DNA extraction were performed using the Instruction manual provided by Qiagen to purify total DNA from animal blood or cells (DNeasy Blood and Tissue Handbook, Spin-Column protocol). All centrifugation steps were performed at room temperature. DNA concentration was quantified twice using a nanodrop 1000 spectrophotometer (PeqLab, Erlangen, Germany). Purified plasmids of p15E-NHR-His and p15E-Link-GFP (1:100 and 1:1000) were used as positive controls. Wt 293T cells were used as a negative control. DNA concentrations were standardized using nuclease free water and 5 μL of DNA sample was added to each reaction mix. DNA from virus producing 293T cells was isolated using the DNeasy Blood and Tissue kit (Qiagen). In order to analyze the expression of cytokines, RNA was isolated using the RNeasy Kit (Qiagen) and DNAse (New England Biolabs, Frankfurt am Main, Germany) treatment to remove cellular DNA.

### 4.12. Polymerase Chain Reaction (PCR), Real-Time PCR and Real-Time RT-PCR

In order to characterize PERV proviruses in virus-producing 293T cells, PCRs using specific primers for the pol and LTR region of PERV (Table 1) were performed using the AmpliTaq DNA polymerase (Applied Biosystems, Waltham, MA, USA): 5 min denaturation at 95 °C, and 45 cycles (15 s 95 °C, 30 s 62 °C, 30 s 72 °C). PCR was performed using a Biometra Thermocycler (Analytik Jena, Jena, Germany). Electrophoresis was performed in a 1.3% agarose gel including a 1 kbp DNA ladder (GeneRuler, Thermo Scientific, Waltham, MA, USA). DNA from a PERV-positive pig was used as a positive control for PCR targeting the *pol* sequence of PERV.

Additionally, two plasmids containing the LTR sequences of PERV-3° and PERV-5° served as positive controls for the characterization of the LTR regions. PERV proviruses in the virus-producing 293T cells were also determined by real-time PCR using specific primers for the pol of PERV (Table 1) and the SensiFAST™ Probe No-ROX Kit (Meridian Bioscience, Cincinnati, OH, USA): 5 min denaturation at 95 °C, and 45 cycles (15 s 95 °C, 30 s 62 °C, 30 s 72 °C). In order to characterize PERV RNA in the supernatant of the virus-producing 293T cells, RT-qPCR was performed using the SensiFAST Probe No-ROX one-step kit (Meridian Bioscience, Cincinnati, OH, USA): 30 min reverse transcription at 50 °C, 5 min denaturation at 95 °C, and 45 cycles (15 s 95 °C, 30 s 62 °C, 30 s 72 °C). qPCR and RT-qPCR were performed using qTOWER^3^ G qPCR-Thermocycler (Analytik Jena, Jena, Germany).

For quantification, a standard curve was built using a synthetic gene block with a partial sequence of PERV pol.

### 4.13. Detection of Cytokine Expression by RT-PCR

In order to analyze expression of IL-6, IL-10, INF-γ, TNF-α, and SEPP1, a RT-PCR for each of the factors was established, using primers and probes as shown in Table 1. RNA was isolated from pooled culture wells, treated with DNAse I, and analyzed using human GAPDH as control: NO-ROX one step kit, 30 min reverse transcription at 50 °C, 5 min denaturation at 95 °C, 45 cycles (10 s 95 °C, 20 s 54 °C, 15 s 72 °C). A qTOWER^3^ qPCR-Thermocycler was used in all experiments. Gene expression was calculated according the 2^−ΔΔCt^ method [64].

### 4.14. Detection of Cytokine Release by ELISA

To detect IL-10, an ELISA for human IL-10 (R & D Biosciences, Minneapolis, MN, USA) was used. Supernatants obtained from co-culture of human PBMCs with transfected and non-transfected 293T cells were used for the assay as described in Section 4.6. Supernatants were collected after 24 h of incubation by centrifuging at 2000× *g* for 10 min. ELISAs were performed in duplicates according to protocols of the supplier: 200 μL of standard, control, and sample supernatant were added per well of the ELISA plate, covered with an adhesive strip, incubated for 2 h at room temperature, and washed with 400 μL of wash buffer using a squirt bottle for a total of 4 washes. A total of 200 μL of human IL-10 antibody conjugate was added to each well and incubated for an hour at room temperature. Washing was repeated, and subsequently, 200 μL of substrate solution was added to each well and incubated for 30 min at room temperature.

Subsequently, 50 μL of stop solution was added to each well. Optical density was determined within 30 min using a microplate reader set to 450 nm. To detect IL-6, an ELISA for human IL-6 (Sigma Aldrich, St. Louis, MI, USA) was used. Supernatants containing proteins were collected after 24 h of incubation by centrifuging at 2000× *g* for 10 min. ELISAs were performed in duplicates according to protocols of the supplier. Standards, buffer solutions, and detection antibodies were prepared as mentioned in manufacturers manual. A volume of 100 μL of supernatant from co-culture of different samples was added in the wells along with the standard. Samples and the standard were incubated overnight at 4 °C with gentle shaking. The next day, solutions were discarded, and wells were washed with 300 μL wash buffer 4 times; 100 μL of biotinylated antibody was added to each well and incubated at room temperature for 60 min with gentle shaking. The biotinylated antibody was removed, wells were washed, and 100 μL of streptavidin solution was added to each well and incubated for 45 min with gentle shaking. Streptavidin was removed from wells, and the plate was washed and incubated with 100 μL TMB one-step substrate reagent for 30 min in the dark with gentle shaking; finally, 50 μL of stop solution was added and optical density was measured immediately at 450 nm on a microplate reader.

### 4.15. Endotoxin Measurement

Although all components to be used for cell culture are endotoxin-free, a measurement of endotoxin was performed using the Pierce chromogenic endotoxin quant kit (Thermo Fisher, Waltham, MA, USA). All tested materials were below the detection limit.

## 5. Conclusions

Using a novel, endotoxin-free cellular system to express the transmembrane envelope protein p15E of PERV, we confirmed the immunosuppressive properties of this molecule. However, these investigations were complicated by the low and fluctuating surface expression of p15E, as assessed by both immunofluorescence and flow cytometry. Further experiments are needed to enhance p15E expression levels in order to obtain more definitive and robust results.

Despite these limitations, an increased release of IL-6 and Il-10, as well as an increased expression of certain cytokines, such as IL-6, IL-10, IFN-α, TNF-α, MMP1, and SEPP1, was observed. Our findings contribute to an understanding of the mechanisms by which transmembrane envelope proteins of retroviruses—including HIV—mediate immunosuppression.

These results are also of particular relevance to the field of xenotransplantation. Expression of such immunosuppressive proteins on the surface of xenografts may help to prevent rejection and reduce the need for pharmacological immunosuppression.

## Figures and Tables

**Table 1:** Primers used for the analysis of cytokine expression and provirus detection.

Primer	Sequence	Reference	Accession Number	Localization
PK 34	5′-AAAGGATGAAAATGCAACCTAACC-3′	Czauderna et al. [40]	Y17012	3–26
PK 26	5′-ACGCACAAGACAAAGACACACGAA-3′			1134–1111
PERV pol fw	5′-CGACTGCCCCAAGGGTTCAA-3′	Yang et al. [41]	HM159246	3568–3587
PERV pol rev	5′-TCTCTCCTGCAAATCTFFGCC-3′			3803–3783
GAPDH fw	5′-GGCCATGCTGGCGCTGAGTAC-3′	Denner et al. [31]	NM 002046.3	364–386
GAPDH rev	5′-TGGTCCACACCCATGACGA-3′			494–512
GAPDH probe	5′-HEX-CTTCACCACCATGGAGAAGGCTGGG-BHQ-1-3′			405–429
IFN-γ fw	5′-TGCAGAGCCAAATTGTCTCC-3′	this manuscript	NM 000619.3	328–347
IFN-γ rev	5′-TGCTTTGCGTTGGACATTCA-3′			502–521
IFN-γ probe	5′-6-FAM-ACCATCAAGGAAGACATGAATGTCAAG-BHQ-1-3′			408–434
IL-6 fw	5′-GGTACATCCTCGACGGCATCT-3′	Denner et al. [31]	NM 000600.3	289–309
IL-6 rev	5′-GTGCCTCTTTGCTGCTTTCAC-3′			349–369
IL-6 probe	5′-6-Fam-TGTTACTCTTGTTACATGTCTCCTTTCTCAGGGCT-BHQ-1-3′			311–345
IL-10 fw	5′-CCACGCTTTCTAGCTGTT-3′	Denner et al. [31]	NM 000572.2	966–983
IL-10 rev	5′-CTCCCTGGTTTCTCTTCCTAA-3′			1058–1078
I-10 probe	5′-6-FAM-TCTTGTCTCTGGGCTT-BHQ-1-3′			1015–1030
MMP1 fw	5′-CATCCAAGCCATATATGGACG-3′	Denner et al. [31]	NM 002421.3	908–928
MMP1 rev	5′-TCTCTTAAAACTGAGAGGTCT-3′			1498–1518
MMP1 probe	5′-6-FAM-CTGGGCTGTTCAGGGACAGAA-BHQ-1-3′			1187–1207
SEPP1 fw	5′-CATGGACATCAGCACCTT-3′	Denner et al. [31]	NM 005410.2	774–459
SEPP1 rev	5′-TCGACAGAGCTTCTTTTG-3′			954–972
SEPP1 probe	5′-6-FAM-AGAATCAGCAACCAGGAGCA-BHQ-1-3′			721–740
TNF-α fw	5′-GAGAAGCAACTACAGACCCC-3′	this manuscript	NM 000594.4	48–67
TNF-α rev	5′-CATGCTTTCAGTGCTCATGG			176–195
TNF-α probe	5′-6-FAM-ACAACCCTCAGACGCCACATCC-BHQ-1-3′			76–97

## Data Availability

The original contributions presented in this study are included in the article. Further inquiries can be directed to the corresponding author.

## Abbreviations

The following abbreviations are used in this manuscript:

BaEV	baboon endogenous retrovirus
BERV	bovine endogenous retrovirus
C-CC	cysteine loop
CKS-17	consensus sequence of the gammaretroviruses
Env	envelope protein
ERV-3	endogenous retrovirus 3
FCN1	ficolin-1
FeLV	feline leukemia virus
FP	fusion peptide
GaLV	gibbon ape leukemia virus
HERV	human endogenous retrovirus
HIV	human immunodeficiency virus
IFN	interferon
IL	interleukin
IP-10	interferon gamma-induced protein 10
Isu	immunosuppressive domain
KoRV	koala retrovirus
Mab-Env1–4	syncytins of Marubya lizards
MCP	monocyte chemoattractant protein
MHC	major histocompatibility complex
MIP	macrophage inflammatory protein
MMP1	matrix metalloproteinase 1
MMTV	mouse mammary tumor virus
MPMV	Mason Pfizer monkey virus
MSD	membrane spanning domain
MuLV	murine leukemia virus
PBMCs	peripheral blood mononuclear cells
PERV	porcine endogenous retrovirus
SEPP1	selenoprotein P, plasma, 1
SHIV	simian–human immunodeficiency virus
SIV cpz	simian immunodeficiency virus chimpanzee
SIV sm	SIV sooty mangabey
SIVagm	SIV African green monkeys
SRV-1	simian retrovirus-1
SU	surface envelope protein
TM	transmembrane envelope protein
TNF	tumor necrosis factor
TREM	triggering receptor expressed on myeloid cells
VEC	vascular endothelial cell
